# Genetic polymorphism and natural selection of circumsporozoite protein in Myanmar *Plasmodium vivax*

**DOI:** 10.1186/s12936-020-03366-7

**Published:** 2020-09-04

**Authors:** Tuấn Cường Võ, Hương Giang Lê, Jung-Mi Kang, Mya Moe, Haung Naw, Moe Kyaw Myint, Jinyoung Lee, Woon-Mok Sohn, Tong-Soo Kim, Byoung-Kuk Na

**Affiliations:** 1 Department of Parasitology and Tropical Medicine, and Institute of Health Sciences, Gyeongsang National University College of Medicine, Jinju, 52727 Republic of Korea; 2 BK21Plus Team for Anti-aging Biotechnology and Industry, Department of Convergence Medical Science, Gyeongsang National University, Jinju, 52727 Republic of Korea; 3 Department of Medical Research Pyin Oo Lwin Branch, Pyin Oo Lwin, Myanmar; 4 Department of Tropical Medicine, Inha University College of Medicine, Incheon, 22212 Republic of Korea

**Keywords:** *Plasmodium vivax*, Circumsporozoite protein, Genetic polymorphism, Natural selection, Myanmar

## Abstract

**Background:**

Circumsporozoite surface protein (CSP) of malaria parasites has been recognized as one of the leading vaccine candidates. Clinical trials of vaccines for vivax malaria incorporating *Plasmodium vivax* CSP (PvCSP) have demonstrated their effectiveness in preventing malaria, at least in part. However, genetic diversity of *pvcsp* in the natural population remains a major concern.

**Methods:**

A total of 171 blood samples collected from patients infected with *Plasmodium vivax* in Myanmar were analysed in this study. The *pvcsp* was amplified by polymerase chain reaction, followed by cloning and sequencing. Polymorphic characteristics and natural selection of *pvcsp* population in Myanmar were analysed using DNASTAR, MEGA6 and DnaSP programs. The polymorphic pattern and natural selection of publicly accessible global *pvcsp* sequences were also comparatively analysed.

**Results:**

Myanmar *pvcsp* sequences were divided into two subtypes VK210 and VK247 comprising 143 and 28 sequences, respectively. The VK210 subtypes showed higher levels of genetic diversity and polymorphism than the VK247 subtypes. The N-terminal non-repeat region of *pvcsp* displayed limited genetic variations in the global population. Different patterns of octapeptide insertion (ANKKAEDA in VK210 and ANKKAGDA in VK247) and tetrapeptide repeat motif (GGNA) were identified in the C-terminal region of global *pvcsp* population. Meanwhile, the central repeat region (CRR) of Myanmar and global *pvcsp*, both in VK210 and VK247 variants, was highly polymorphic. The high level of genetic diversity in the CRR has been attributed to the different numbers, types and combinations of peptide repeat motifs (PRMs). Interestingly, 27 and 5 novel PRMs were found in Myanmar VK210 and VK247 variants, respectively.

**Conclusion:**

Comparative analysis of the global *pvcsp* population suggests a complex genetic profile of *pvcsp* in the global population. These results widen understanding of the genetic make-up of *pvcsp* in the global *P. vivax* population and provide valuable information for the development of a vaccine based on PvCSP.

## Background

Malaria caused by *Plasmodium* species has threatened human health since ancient time [1]. The mortality and morbidity of malaria has greatly decreased in recent years; however, an estimated 219 million cases and 435,000 deaths due to malaria were reported globally in 2017 [2]. Among five human malaria parasites, *Plasmodium vivax* is the most prevalent species outside of Africa. More than 7.5 million cases of malaria were caused by the parasite, which accounted for 56% of total malaria cases in South East Asia [2]. *Plasmodium vivax* has been linked to benign malarial infection due to its mild clinical manifestations compared with *Plasmodium falciparum*; however, concerns that *P. vivax* also causes serious clinical illnesses and even death have increased [3, 4]. The emergence and spread of drug-resistant strains also increases the burden of the parasite [5, 6]. Furthermore, the liver stages in the life cycle of *P. vivax*, known as hypnozoites, can remain dormant and survive for weeks to months in liver cells, and be reactivated [7]. This unique biological characteristic is a challenge in the control and elimination of this parasite. Therefore, the development of an effective vaccine is imperative for effective control and elimination of the parasite.

Circumsporozoite protein (CSP) is the most abundantly expressed protein on the surface of sporozoites. CSP plays multiple and crucial roles in the development, migration and invasion of sporozoites into hepatocytes [8]. Thus, this protein has been studied extensively as one of the most promising vaccine candidates. The gene encoding CSP consists of three distinct regions: a conserved non-repeat N-terminal region, a highly polymorphic central repeat region (CRR), and a conserved non-repeat C-terminal region (Additional file 1: Figure S1). The CRR of *P. vivax* CSP (PvCSP) consists of peptide repeat motifs (PRMs). Based on the composition of one of the three major PRMs in the CRR, *pvcsp* is classified into three allelic variants: VK210, VK247 and *P. vivax*-like. The two most abundant alleles include VK210 and VK247, which carry nonapeptide repeat motifs GDRA(D/A)GQPA and ANGAG(N/D)QPG, respectively [9]. Meanwhile, the *P. vivax*-like variant contains APGANQ(E/G)GAA motifs in the CRR [10].

Although no licensed vaccine against vivax malaria is currently available, a few notable advances in the development of a potential vaccine have been reported. A vivax malaria subunit vaccine named VMP001 has been clinically tested. This vaccine uses a chimeric recombinant protein containing repeat sequences of two major alleles of *pvcsp*, including VK210 and VK247 [11]. The phase 1 trial with VMP001 showed a significant delay in parasitaemia, even though vaccination did not induce fully sterilizing protective immunity [12]. However, the geographical variation in the genetic make-up of *pvcsp* population may influence the effectiveness of PvCSP-based vaccine, which may be a barrier for the development of a universal vaccine. Therefore, a comprehensive analysis of genetic diversity and structure of the global *pvcsp* population is necessary. In this study, genetic polymorphism and natural selection of Myanmar *pvcsp* has been examined. A comparative analysis of global *pvcsp* was also conducted to obtain a deeper insight into the genetic nature of *pvcsp* in the global *P. vivax* population.

## Methods

### Blood samples

**Fig. 1:**
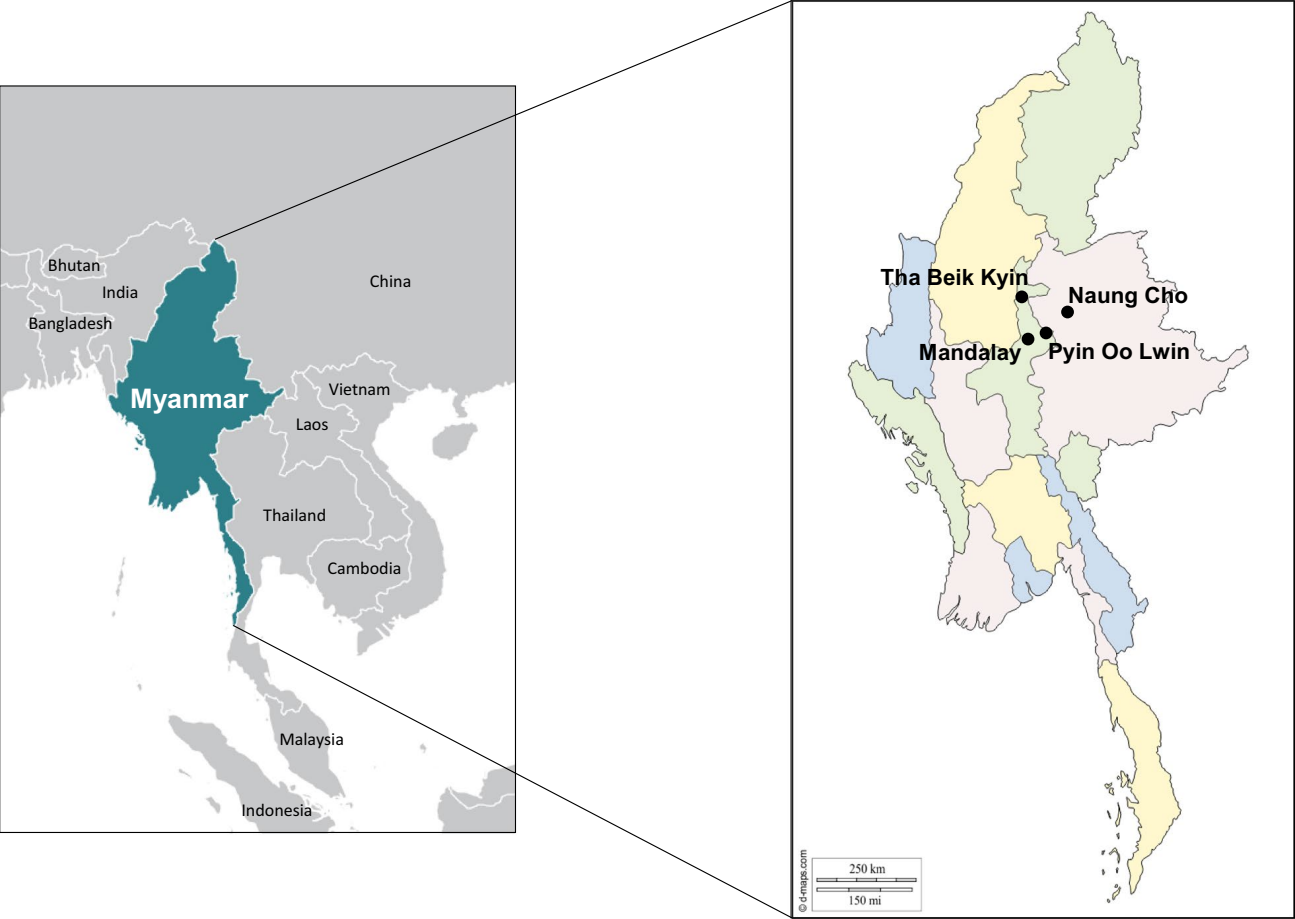
Map of the study sites. Blood samples were collected from *P. vivax* infected patients, who resided in villages or townships in Naung Cho, Pyin Oo Lwin and Tha Beik Kyin, Myanmar and used in this study

One hundred seventy-one blood samples from *P. vivax*-infected Myanmar patients were used in this study. The blood samples were collected from the patients in field surveys in towns and villages in Naung Cho, Pyin Oo Lwin, and Tha Beik Kyin in Upper Myanmar during 2013 to 2015 (Fig. 1). The age range of patients was 13–62 years, with median age of 28.4 years. Initial screening for malaria infection was done by microscopic examination of thin and thick blood smears. Finger-prick blood samples were taken from the *P. vivax*-infected patients before drug treatment and spotted on Whatman 3 MM filter paper (GE Healthcare, Maidstone, UK) for confirmation by species-specific polymerase chain reaction (PCR) targeting 18S ribosomal RNA (rRNA) gene [13, 14]. Informed consent was obtained from all of the patients before blood collection. The study protocol was reviewed and approved by either the Ethics committee of the Ministry of Health, Myanmar (97/Ethics 2015) and the Biomedical Research Ethics Review Board of Inha University School of Medicine, Republic of Korea (INHA 15-013).

### Genomic DNA extraction and amplification of *pvcsp*

Genomic DNA was extracted from dried blood spots using QIAamp DNA Blood Kit (Qiagen, Hilden, Germany) following the manufacturer’s protocol. Amplification of *pvcsp* gene was performed with primers by using nested PCR. The primers for first round PCR were 5ʹ-ATGTAGATCTGTCCAAGGCCATAAA-3ʹ and 5ʹ-AATTGAATAATGCTAGGACTAACAATATG-3ʹ. The thermal cycling parameters for primary PCR were as follows: one cycle of initial denaturation at 95 °C for 5 min, 25 cycles of 94 °C for 1 min, annealing at 58 °C for 2 min and extension at 72 °C for 2 min, followed by a final extension at 72 °C for 5 min. The nested PCR was performed with primers, 5ʹ-GCAGAACCAAAAAATCCACGTGAAAATAAG-3ʹ and 5ʹ-CCAACGGTAGCTCTAACTTTATCTAGGTAT-3ʹ, and similar amplification condition except the annealing temperature was 68 °C. Ex *Taq* DNA polymerase (Takara, Otsu, Japan) with proof-reading activity was used in all PCR steps to minimize the nucleotide mismatching during the amplification. Each PCR product was analysed by electrophoresis on 2% agarose gel. The resulting PCR product was extracted from the gel and was cloned into T&A cloning vector (Real Biotech Corporation, Banqiao City, Taiwan). Each ligation mixture was transformed into *Escherichia coli* DH5α competent cells. To identify positive clones with appropriate insert, colony PCR with nested PCR primers was performed. The nucleotide sequences of the cloned *pvcsp* were analysed by the Sanger method with M13 forward and reverse primers. Plasmids from at least two independent clones from each transformation mixture were analysed to confirm the sequence accuracy. These nucleotide sequences analysed in this study were deposited at GenBank under the accession numbers MN821829–MN821999.

### Analyses of genetic diversity and natural selection in Myanmar *pvcsp*

The nucleotide and deduced amino acid sequences of Myanmar *pvcsp* were analysed using Editseq and Seqman in the DNASTAR package (DNASTAR, Madison, WI, USA). Two major variants of *pvcsp* sequences, Salvador I (Sal I; GU339059) and Papua New Guinea (PNG; M69059), were used as reference sequences to analyse Myanmar *pvcsp* sequences. The values of number of segregating sites (S), haplotypes (H), haplotype diversity (Hd), nucleotide diversity (π), and average number of pair-wise nucleotide differences within a population (*K*) were calculated with DnaSP ver. 5.10.00 [15]. The rate of synonymous (dS) and nonsynonymous (dN) substitutions were calculated and compared using the Z-test (*P *< 0.05) with MEGA6 program [16] using Nei and Gojobori’s method [17] with the Juke and Cantor (JC) correction of 1000 bootstrap replications. Based on the acquired vales, the dN–dS value was calculated. Positive value for dN–dS imply to positive natural selection whereas negative value correspond to negative or purifying natural selection [18]. Tajima’s D test, Fu and Li’s D and F statistics analysis was performed using DnaSP ver. 5.10.00 [15] to evaluate the neutral theory of natural selection. An excess of high-frequency variation is consistent with balancing selection and is indicated by a positive Tajima’s D and/or Fu and Li’s D [19, 20]. A negative value of Tajima’s D and/or Fu and Li’s D indicates an excess of rare alleles, which may result from a recent selective sweep or purifying selection.

### Genetic diversity and natural of *pvcsp* among the global *P. vivax* population

The genetic diversity of *pvcsp* among the global *P. vivax* population was also analysed. The *pvcsp* sequences deposited in public database were used in this study; Cambodia (*n* = 41), India (*n* = 79), Iran (*n* = 50), South Korea (*n* = 39), Brazil (*n* = 41), Mexico (*n* = 19), Colombia (*n* = 25), Sudan (*n* = 30), and Vanuatu (*n* = 21) (Additional file 2: Table S1). Genetic polymorphism and test of neutrality were examined for each *pvcsp* population with DnaSP ver 5.10.00 [15] and MEGA6 [16] as described above. To analyse the polymorphic patterns of the N- and C-terminal non-repeat regions in global *pvcsp*, a logo plot was constructed for each population using the WebLogo program (https://weblogo.berkeley.edu/logo.cgi).

## Results

### Amplification and sequence analysis of Myanmar *pvcsp*

A total of 171 Myanmar *pvcsp* genes were successfully amplified form the genomic DNA samples used in this study. The size of the amplified *pvcsp* genes ranged from 0.5 to 1.3 kb. Sequence analysis of the amplified *pvcsp* genes revealed only two variants of *pvcsp*, VK210 and VK247, in Myanmar *pvcsp*, but not the *P. vivax*-like variant. The VK210 variants were prevalent (*n* = 143, 83.6%) and the frequency of VK247 variants was 16.4% (*n* = 28). No mixed infection with the two different variants was detected.

### Genetic diversity of the N-terminal non-repeat region of Myanmar *pvcsp*

**Fig. 2:**
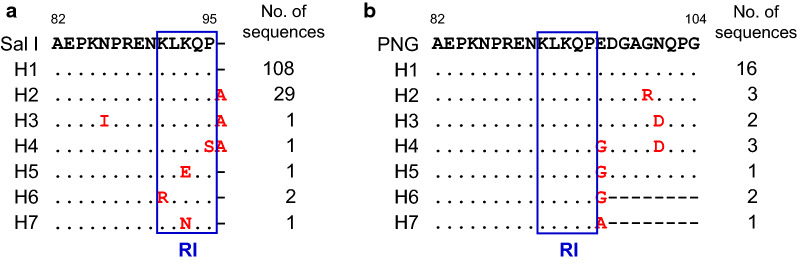
Polymorphic patterns of the N-terminal non-repeat region of Myanmar *pvcsp* sequences. **a** VK210 variants. Seven distinct haplotypes of the N-terminal non-repeat region was identified in 143 Myanmar VK210 sequences. The dots represent residues identical to the reference sequence of Sal I (GU339059). Amino acid changes at particular amino acid positions are indicated as red. The dashes represent gaps to maximize the alignment. **b** VK247 variants. Seven distinct haplotypes of the N-terminal non-repeat region was identified in 28 Myanmar VK247 sequences. The dots represent residues identical to the reference sequence of PNG (M69059). Amino acid changes at particular amino acid positions are indicated as red. The dashes represent gaps to maximize the alignment. RI means KLKQP motif that is involved in the sporozoite invasion of mosquito salivary gland and in binding to hepatocytes prior to invasion. The total number of sequences for each hapoltype is listed in the right panel

The N-terminal non-repeat region of Myanmar *pvcsp* showed a limited range of genetic diversity. Alignment of the deduced amino acid sequences of Myanmar *pvcsp* revealed 7 distinct haplotypes of VK210 variants and 7 haplotypes of VK247 variants (Fig. 2). The N-terminal non-repeat region of Myanmar VK210 variants was highly conserved. Compared with Sal I (GU339059) sequence, only few amino acid substitutions was found in the latter portion of the N-terminal non-repeat region. Haplotype 1, which was identical to Sal I sequence, was predominant (*n* = 108, 75.5%). An alanine insertion at the end of the RI conserved motif (KLKQP) was identified in haplotypes 2, 3 and 4. The N86I was found in haplotype 3. Two dimorphic (K91R and P95S) and one trimorphic (K93E/N) amino acid changes were observed in the RI motif of haplotypes 4, 5, 6, and 7 (Fig. 2a). Changes in the amino acid sequences were also identified in VK247 variants of Myanmar *pvcsp*. Haplotype 1, which shared the same sequence with the PNG (M69059) sequence, was the most prevalent, accounting for 57.1% of 28 Myanmar VK247 variants. All amino acid changes identified in Myanmar VK247 variants were all dimorphic (E96G/A, G99R, and N100D). Furthermore, eight amino acids (^97^DGAGNQPG^104^) were not detected in haplotypes 6 and 7 (Fig. 2b).

### Genetic polymorphisms of the N-terminal non-repeat region in global *pvcsp*

**Fig. 3:**
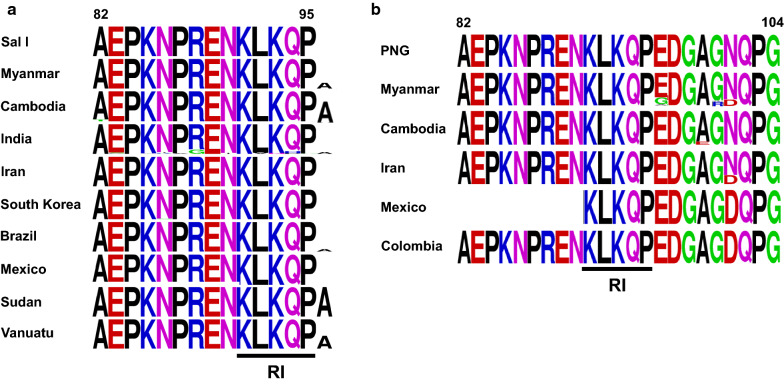
Comparative analysis of polymorphic patterns of N-terminal non-repeat region in global *pvcsp*. **a** Global VK210 variants. **b** Global VK247 variants. The pattern of amino acid changes differed by country or geographic continent. A logo plot was constructed for each *pvcsp* population using the WebLogo program. Sal I, Salvador I (GU339059); PNG, Papua New Guinea (M69059). RI region was marked as an underline

Overall genetic polymorphisms of the N-terminal non-repeat region in the global *pvcsp* population were analysed. A comparative analysis of the region revealed that the region is relatively well-conserved in the global *pvcsp*. Alanine insertion at the end of the RI in the VK210 variants was the major variation identified in the global *pvcsp*, but the prevalence of the insertion differed by geographically (Fig. 3a). The *pvcsp* from Sudan showed 100% alanine insertion followed by *pvcsp* from Cambodia (67.7%), Vanuatu (47.6%), Myanmar (21.7%), Brazil (9.8%), and India (7.6%). No alanine insertion was found in the VK210 sequences identified in Iran, South Korea, and Mexico. Indian VK210 variants showed the highest genetic diversity with amino acid substitutions at 10 positions including E83K/G, K85T/Q, N86K/Y/I, P87A, R88G, N90I, L92V, K93N, Q94H/P and P95G, even though their frequencies were low, less than 15%. Meanwhile, A82T, N86I, N86S, K91R, P95S, and K93E/N showed uneven geographic distribution in the global VK210 variants with very low frequencies. The N-terminal non-repeat region of global VK247 variants were also well-conserved, although low frequencies of uneven amino acid changes were identified (Fig. 3b). The most remarkable variation involved N100D, which was observed in VK247 variants from Colombia (100%), Mexico (100%), Iran (27.3%), and Myanmar (17.9%). The amino acid changes such as E96G/A, A99E, G100R, and N101D showed uneven geographic distribution with low frequencies.

### Polymorphic pattern of the CRR in Myanmar *pvcsp*

**Fig. 4:**
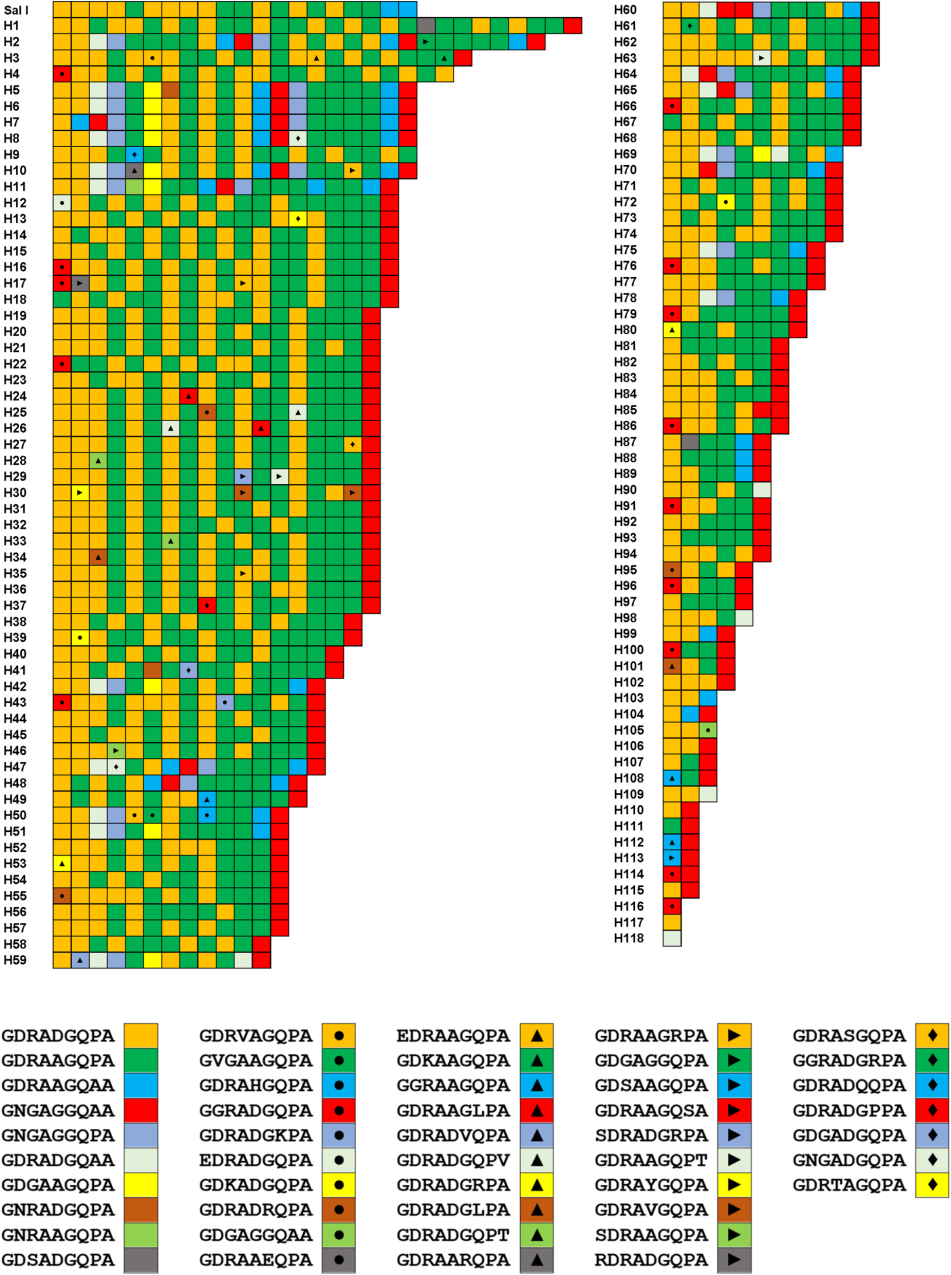
Genetic polymorphism in the central repeat region (CRR) of Myanmar VK210 variants. Sequence alignment revealed that the CRR of Myanmar VK210 variants showed polymorphic characters with 118 distinct haplotypes. A total of 47 different types of peptide repeat motifs (PRMs) were identified in CRR of Myanmar VK210 variants. Differences in numbers, types, and combinations of PRMs produced the high levels of genetic diversity of the CRR in Myanmar VK210 variants

**Fig. 5:**
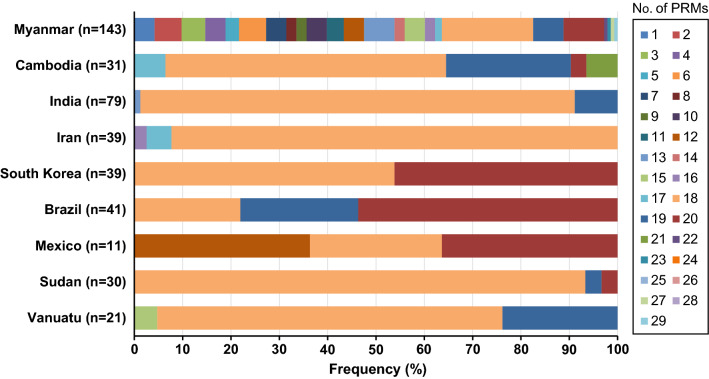
Frequency comparison of peptide repeat motifs (PRMs) in the central repeat region (CRR) of global VK210 variants. The numbers of PRMs differed in the global VK210 varaints

The CRR of Myanmar *pvcsp* showed extreme diversity in both VK210 and VK247 variants. A total of 118 and 23 haplotypes were identified in VK210 and VK247 variants, respectively. As expected, the greatest diversity of Myanmar *pvcsp* CRR was mainly attributed to differences in numbers, types, and arrangements of PRMs in each haplotype. A total of 47 different types of PRMs have been identified in the CRR of Myanmar VK210 variants (Fig. 4). Among these PRMs, two major types GDRADGQPA and GDRAAGQPA were the dominant ones for VK210 variants. Twenty-seven novel PRMs including GDRVAGQPA, GDRAHGQPA, GDRADGKPA, GDRADRQPA, GDGAGGQAA, EDRAAGQPA, GDKAAGQPA, GDRAAGLPA, GDRADVQPA, GDRADGQPV, GDRADGRPA, GDRADGLPA, GDRADGQPT, GDRAARQPA, GDRAAGRPA, GDGAGGQPA, GDRAAGQSA, SDRADGRPA, GDRAAGQPT, GDRAYGQPA, SDRAAGQPA, RDRADGQPA, GDRASGQPA, GGRADGRPA, GDRADQQPA, GDRADGPPA and GNGADGQPA, which were not previously reported, were found in Myanmar VK210 variants. Interestingly, 109 haplotypes out of 118 VK210 variants were terminated with GNGAGGQAA motif. The number of PRMs consisting CRR of Myanmar VK210 haplotypes varied from 1 to 29. Sequences with 18 PRMs were the most prevalent accounting for 18.9% of 143 Myanmar VK210 sequences (Fig. 5).

**Fig. 6:**
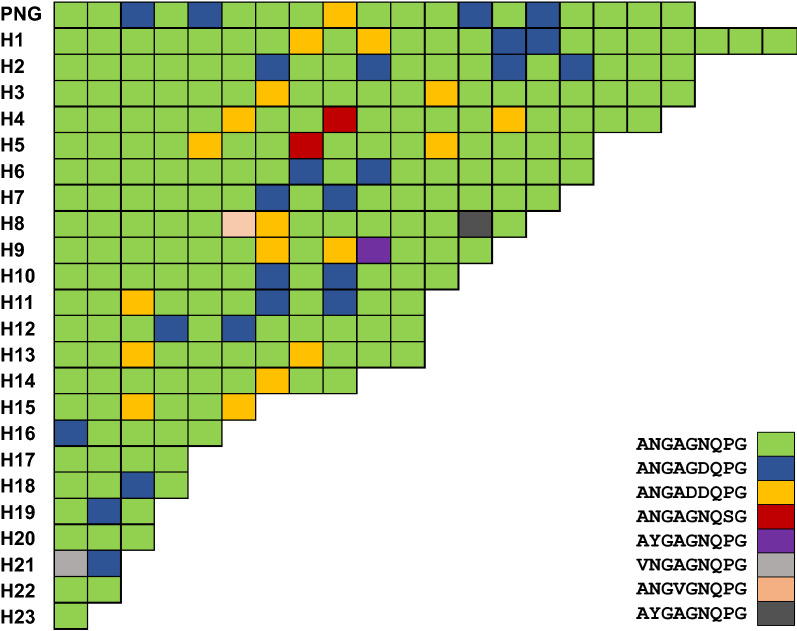
Genetic polymorphism in the central repeat rsegion (CRR) of Myanmar VK247 varaints. Sequence alignment revealed that the CRR of Myanmar VK247 variants showed polymorphic characters with 23 distinct haplotypes. A total of 8 different types of peptide repeat motifs (PRMs) were identified in CRR of Myanmar VK247 variants. Differences in numbers, types, and combinations of PRMs produced the high levels of genetic diversity of the CRR in Myanmar VK247 variants

**Fig. 7:**
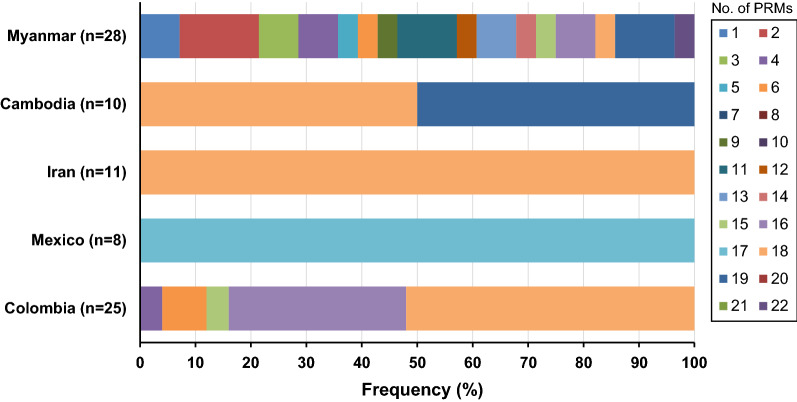
Frequency comparison of peptide repeat motifs (PRMs) in the central repeat region of global VK247 variants. The numbers of PRMs differed in the global VK210 varaints

Compared with global VK210 variants, the CRR of Myanmar VK210 variants showed a high level of length polymorphisms. The VK210 variants from other countries analysed in this study showed only a few (2 to 5) variations in length of polymorphisms in CRR; however, the CRR of Myanmar VK210 variants had 24 different length polymorphisms consistent with various types and different compositions of PRMs. The overall genetic diversity in CRR of Myanmar VK247 variants was much lower than that of Myanmar VK210 variants. A total of 23 VK247 haplotypes, each CRR comprising different numbers and combinations of 8 types of PRMs, were identified (Fig. 6). Five of 8 PRMs, ANGAGNQSG, AYGAGNQPG, VNGAGNQPG, ANGVGNQPG and AYGAGNQPG, identified in Myanmar VK247 variants were novel ones that have not reported previously. The CRR of Myanmar VK247 variants carried different numbers of PRMs ranging from 1 to 22, and the CRR with 2 PRMs was the most prevalent (14.3%) (Fig. 7). Similar to VK210 variants, the Myanmar VK247 variants also displayed higher levels of diversity than the VK247 variants identified from other geographical origins. Sixteen different size polymorphisms of CRR were identified in Myanmar VK247 variants, whereas less size variations (1 to 5) were found in CRR of all other countries analysed in this study.

### Genetic diversity in the C-terminal non-repeat region of Myanmar and global *pvcsp*

**Fig. 8:**
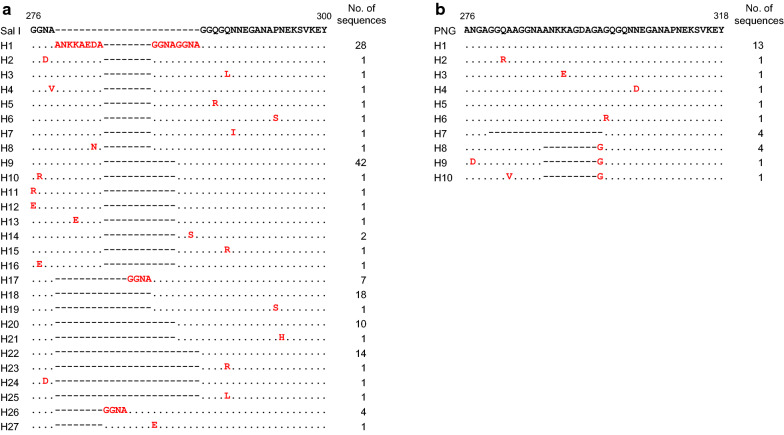
Genetic polymorphism of the C-terminal non-repeat region of Myanmar *pvcsp* sequences. **a** VK210 variants. A total of 27 distinct haplotypes of the C-terminal non-repeat region was identified in 143 Myanmar VK210 sequences. The dots represent residues identical to the reference sequence of Sal I (GU339059). Amino acid changes at particular amino acid positions are indicated as red. The dashes represent gaps to maximize the alignment. **b** VK247 variants. Ten distinct haplotypes of the C-terminal non-repeat region was identified in 28 Myanmar VK247 sequences. The dots represent residues identical to the reference sequence of PNG (M69059). Amino acid changes at particular amino acid positions are indicated as red. The dashes represent gaps to maximize the alignment. The total number of sequences for each hapoltype is listed in the right panel

**Fig. 9:**
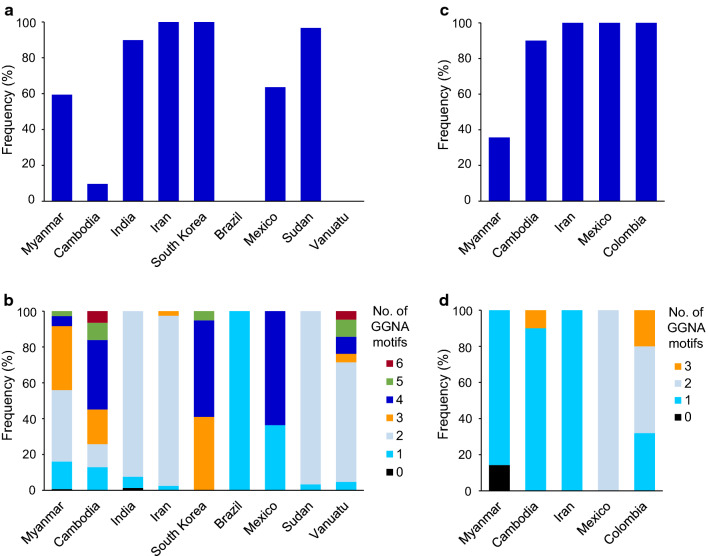
Frequency of octa-peptide insertion and GGNA motif in the C-terminal non-repeat region of global *pvcsp*. **a** Frequency of ANKKAEDA octa-peptide insertion in the C-terminal non-repeat region of global VK210 variants. **b** Frequency of GGNA repeat motifs in the C-terminal non-repeat region of global VK210 variants. **c** Frequency of ANKKAGDA insertion in the C- terminal region of global VK247 variants. **d**) Frequency of GGNA repeat motifs in the C- terminal region of global VK247 variants

Sequence analysis of the C-terminal non-repeat region of Myanmar VK210 variants revealed 27 distinct haplotypes (Fig. 8a). These sequence diversities were attributed to differences in the arrangement of ANKKAEDA octapeptide insertion and GGNA tetrapeptide repeat motifs with different amino acid substitutions throughout the region. The sequence of haplotype 22 was identical with Sal I (GU339059), and accounted for 9.8% of all the VK210 sequences. The octapeptide insertions were observed in haplotypes 1 to 16. All the inserted sequences in these 16 haplotypes were ANKKAEDA except for haplotypes 8 and 13, which contained ANKKAENA and ANKEAENA, respectively. The C-terminal non-repeat region of Myanmar VK247 variants showed a lower level of genetic diversity than that of Myanmar VK210 variants (Fig. 8b). A total of 10 haplotypes were identified, and haplotype 1, which carried a sequence identical to that of the reference sequence of PNG (M69059), was the most prevalent haplotype with a frequency of 46.4%. The most noteworthy polymorphic characteristics identified in the C-terminal non-repeat region of Myanmar VK247 variants were the deletions of GGQAAGGNAANKKAGDAG in haplotype 7 and ANKKAGDAG in haplotypes 8, 9 and 10. Analysis of sequence polymorphisms in the C-terminal non-repeat region of the global *pvcsp* suggested a high level of genetic diversity. Among VK210 variants, the frequency of ANKKAEDA insertion differed by country (Fig. 9a). All sequences from Iran and South Korea VK210 variants contained an octapeptide insertion, but the frequencies in VK210 variants from Sudan, India, Mexico and Cambodia were 96.7, 89.9, 63.6 and 9.7%, respectively. Interestingly, no insertion of the octapeptide was identified in VK210 variants from Brazil and Vanuatu. The numbers of GGNA motifs in the C-terminal non-repeat region of global VK210 variants also differed by country (Fig. 9b). The number of repeated GGNA motifs found in global VK210 variants ranged from 0 to 6. Similar to VK210 variants, the presence of ANKKAGDAG octapeptide insertion and the number of GGNA motifs also differed in the C-terminal non-repeat region of global VK247 variants. The frequency of ANKKAGDAG octapeptide insertion was high in VK247 variants isolated from Cambodia, Iran, Mexico, and Colombia, but was low in Myanmar VK247 variants (Fig. 9c). The number of GGNA motifs found in the C-terminal non-repeat region of global VK247 variants ranged from 0 to 3 (Fig. 9d). The number of GGNA motifs in global VK247 also differed by country.

### Nucleotide diversity and natural selection in the N- and C-terminal non-repeat regions of Myanmar *pvcsp*

Since the CRR of Myanmar *pvcsp* sequences showed a high degree of length polymorphisms, the nucleotide diversity and genetic differentiation of the N- and C-terminal non-repeat regions were analysed separately by omitting the CRR. In the N-terminal non-repeat region of Myanmar VK210 variants, the average number of nucleotide differences (*K*), overall haplotype diversity (Hd), and nucleotide diversity (π) were 0.098, 0.096 ± 0.034, and 0.0024 ± 0.0009, respectively (Table 1). The estimated dN–dS value in the N-terminal non-repeat regions was 0.0008. These results suggested that the N-terminal non-repeat region of Myanmar VK210 variants was under positive natural selection. Meanwhile, the average number of nucleotide differences (*K*), the overall haplotype diversity (Hd), and the nucleotide diversity (π) for the C-terminal non-repeat region of Myanmar VK210 variants were 0.209, 0.186 ± 0.044, and 0.0035 ± 0.0009, respectively (Table 1). The dN–dS value was –0.0035. These findings indicated that the C-terminal region was influenced by negative natural selection. The Tajima’s D test was also performed to further elucidate the effect of natural selection on the N- and C-terminal non-repeat regions in Myanmar VK210 variants. Tajima’s D values for the N- and C-terminal non-repeat regions were –1.9359 (*P* < 0.05) and –2.2251 (*P* < 0.01), respectively (Table 1). The Fu and Li’s D and F values of these regions also showed negative values. In the Myanmar VK247 variants, the average number of nucleotide differences (*K*), overall haplotype diversity (Hd) and nucleotide diversity (π) of the N-terminal non-repeat region were 0.405, 0.405 ± 0.094 and 0.0090 ± 0.0021, respectively (Table 1). These values for the C-terminal non-repeat region were 0.495, 0.331 ± 0.114 and 0.0067 ± 0.0027, respectively. The dN–dS values of the N- and C-terminal non-repeat regions of Myanmar VK247 variants were 0.0117 and –0.0150, respectively (Table 1). The Tajima’s D values were –0.4445 (*P* > 0.1) and –1.9719 (*P* < 0.05) for the N- and C-terminal non-repeat regions, respectively (Table 1). The Fu and Li’s D and F values for both regions were all negative.

**Table 1:** Genetic polymorphism and tests of neutrality in the N-terminal and C-terminal regions of Myanmar *pvcsp*

Variant	Region	n	K	S	Eta	H	Hd ± SD	π ± SD	dN-dS	Tajima’s D^*P* value^	Fu and Li’s D^*P* value^	Fu and Li’s F^*P* value^
VK210	N-terminal	143	0.098	5	6	7	0.096 ± 0.034	0.0024 ± 0.0009	0.0008	− 1.9359^c^	− 3.9086^b^	− 3.8409^b^
	C-terminal	143	0.209	10	11	11	0.186 ± 0.044	0.0035 ± 0.0009	−0.0035	− 2.2251^d^	− 3.5089^b^	− 3.6329^b^
VK247	N-terminal	28	0.405	1	2	3	0.405 ± 0.094	0.0090 ± 0.0021	0.0117	− 0.4445^a^	− 0.7114^a^	− 0.7369^a^
	C-terminal	28	0.495	6	6	6	0.331 ± 0.114	0.0067 ± 0.0027	− 0.0150	− 1.9719^c^	− 2.5946^c^	− 2.8039^c^

*n* number of analysed sequences, *K* average number of nucleotide differences, *S* number of segregating sites, *Eta* total number of mutations, *H* number of haplotypes, *Hd* haplotype diversity, *π* observed average pairwise nucleotide diversity, *SD* standard deviation, *dN* rate of non-synonymous mutations, *dS* rate of synonymous mutations

^a^*P *> 0.1

^b^*P* < 0.02

^c^*P* < 0.05

^d^*P* < 0.01

### Nucleotide diversity and natural selection in the N- and C-terminal non-repeat regions of global VK210 variants

To further examine the nucleotide diversity and natural selection in the global *pvcsp* population, the nucleotide diversity of the N- and C-terminal non-repeat regions of global *pvcsp* was analysed. For the N-terminal non-repeat region of VK210 variants, nucleotide diversity and pattern of natural selection were differed by country (Table 2). VK210 variants from India showed the highest nucleotide diversity; the values of *K*, Hd, and π were 1.972, 0.661 ± 0.063, and 0.0470 ± 0.0074, respectively. The N-terminal non-repeat region of Cambodia VK210 variants also showed relatively high nucleotide diversity comparable to Myanmar VK210 variants. Substantial nucleotide diversity was found in the VK210 variants from South Korea and Brazil. However, VK210 variants from Iran, Mexico, Sudan, and Vanuatu were genetically well-conserved. The N-terminal non-repeat region of VK210 variants showed different patterns of natural selection by country. The dN–dS value was positive for VK210 variants from Myanmar, Cambodia, and South Korea, which indicated positive natural selection may occur in the region. Meanwhile, the values for VK210 variants from India and Brazil were negative, suggesting negative selection. The values of Tajima’s D for all VK210 variants derived from Myanmar, Cambodia, India, South Korea, and Brazil were negative, indicating that they were under purifying selection. The C-terminal non-repeat region of global VK210 variants also revealed nucleotide diversity and pattern of natural selection (Table 2). Indian VK210 variants showed the greatest nucleotide diversity with *K*, Hd, and π values of were 0.276, 0.123 ± 0.051, and 0.0043 ± 0.0020, respectively. Meanwhile, no nucleotide diversity was detected in VK210 variants from Cambodia, Iran, Brazil, Mexico, and Vanuatu. Similar to the N-terminal non-repeat region, the C-terminal non-repeat region of global VK210 was also under the effect of purifying selection based on the negative values of Tajima’s D. The values of Fu and Li’s D and Fu and Li’s F were also negative for the C-terminal region of VK210 variants from Myanmar, India, South Korea, and Sudan.

**Table 2:** Genetic polymorphism and tests of neutrality in the N-terminal and C-terminal non-repeat regions of global VK210 variants

Region	Country	n	*K*	S	Eta	H	Hd ± SD	π ± SD	dN-dS	Tajima’s D^*P* value^	Fu and Li’s D^*P* value^	Fu and Li’s F^*P* value^
N-terminal	Myanmar	143	0.098	5	6	7	0.096 ± 0.034	0.0024 ± 0.0009	0.0008	− 1.9359^c^	− 3.9086^b^	− 3.8409^b^
	Cambodia	31	0.125	1	1	2	0.125 ± 0.077	0.0030 ± 0.0018	0.0039	− 0.7737^a^	0.5907^a^	0.2450^a^
	India	79	1.972	23	28	28	0.661 ± 0.063	0.0470 ± 0.0074	− 0.0909	− 2.0261^c^	− 1.3952^a^	− 1.9514^e^
	Iran	39	0	0	0	1	0	0	0	0	0	0
	South Korea	39	0.051	1	1	2	0.051 ± 0.048	0.0012 ± 0.0011	0.0016	− 1.1264^a^	− 1.7662^a^	− 1.8293^a^
	Brazil	41	0.049	1	1	2	0.049 ± 0.046	0.0012 ± 0.0011	− 0.0059	− 1.1219^a^	− 1.7816^a^	− 1.8406^a^
	Mexico	11	0	0	0	1	0	0	0	0	0	0
	Sudan	30	0	0	0	1	0	0	0	0	0	0
	Vanuatu	21	0	0	0	1	0	0	0	0	0	0
C-terminal	Myanmar	143	0.209	10	11	11	0.186 ± 0.044	0.0035 ± 0.0009	− 0.0035	− 2.2251^d^	− 3.5089^b^	− 3.6329^b^
	Cambodia	31	0	0	0	1	0	0	0	0	0	0
	India	79	0.276	8	9	6	0.123 ± 0.051	0.0043 ± 0.0020	− 0.0149	− 2.1953^d^	− 4.4419^b^	− 4.3524^b^
	Iran	39	0	0	0	1	0	0	0	0	0	0
	South Korea	39	0.103	2	2	3	0.101 ± 0.065	0.0008 ± 0.0005	− 0.0037	− 1.4889^a^	− 2.4148^e^	− 2.4864^e^
	Brazil	41	0	0	0	1	0	0	0	0	0	0
	Mexico	11	0	0	0	1	0	0	0	0	0	0
	Sudan	30	0.067	1	1	2	0.067 ± 0.061	0.0009 ± 0.0008	− 0.0041	− 1.1470^a^	− 1.6821^a^	− 1.7655^a^
	Vanuatu	21	0	0	0	1	0	0	0	0	0	0

*n* number of analysed sequences, *K* avarage number of nucleotide differences, *S* number of segregating sites, *Eta* total number of mutations, *H* number of haplotypes, *Hd* haplotype diversity, *π* observed average pairwise nucleotide diversity, *SD* standard deviation, *dN* rate of non-synonymous mutations, *dS* rate of synonymous mutations

^a^*P *> 0.1

^b^*P *< 0.02

^c^*P *< 0.05

^d^*P *< 0.01

^e^0.05 < *P *< 0.1

### Nucleotide diversity and natural selection in the N-terminal and C-terminal non-repeat regions of global VK247 variants

The nucleotide diversity and natural selection in the global VK247 variants were analysed (Table 3). Analysis of the N-terminal non-repeat region of VK247 variants revealed the greatest nucleotide diversity in VK247 variants from Iran, with the values of *K*, Hd, and π of 1.309, 0.436 ± 0.133, and 0.0190 ± 0.0058, respectively. VK247 variants from Cambodia also revealed nucleotide diversity in the region. However, no nucleotide diversity was found in VK247 variants from Mexico and Colombia. The N-terminal non-repeat region of VK247 variants from Iran showed negative dN–dS (–0.0543) and positive Tajima’s D (0.9518), Fu and Li’s D (1.1271), and Fu and Li’s F (1.2185). However, Myanmar and Cambodia VK247 variants revealed positive values for dN–dS and negative values for Tajima’s D, Fu and Li’s D, and Fu and Li’s F. The C-terminal non-repeat region of VK267 variants from Cambodia and Colombia also showed nucleotide diversity, which was lower than that of Myanmar VK247 variants. Similar to Myanmar VK247 variants, the C-terminal non-repeat region of Cambodia VK247 variants showed a negative values of dN–dS (–0.0172), Tajima’s D (–1.4009), Fu and Li’s D (–1.5866), and Fu and Li’s F (–1.7190). However, the dN–dS of Colombia variants was estimated to be positive (0.0017), although the values of Tajima’s D, Fu and Li’s D, and Fu and Li’s F were negative.

**Table 3:** Genetic polymorphism and tests of neutrality in N-terminal and C-terminal regions of global *pvcsp* VK247 variants

Region	Country	n	*K*	S	Eta	H	Hd ± SD	π ± SD	dN-dS	Tajima’s D^*P* value^	Fu andLi’s D^*P *value^	Fu and Li’s F^*P *value^
N-terminal	Myanmar	28	0.405	1	2	3	0.405 ± 0.094	0.0090 ± 0.0021	0.0117	− 0.4445^a^	− 0.7114^a^	− 0.7369^a^
	Cambodia	10	0.2	1	1	2	0.200 ± 0.154	0.0029 ± 0.0022	0.0038	− 1.1117^a^	− 1.2434^a^	− 1.3467^a^
	Iran	11	1.309	3	3	2	0.436 ± 0.133	0.0190 ± 0.0058	− 0.0543	0.9518^a^	1.1271^a^	1.2185^a^
	Mexico	8	0	0	0	1	0	0	0	0	0	0
	Colombia	25	0	0	0	1	0	0	0	0	0	0
C-terminal	Myanmar	28	0.495	6	6	6	0.331 ± 0.114	0.0067 ± 0.0027	− 0.0150	− 1.9719^c^	− 2.5946^c^	− 2.8039^c^
	Cambodia	10	0.400	2	2	2	0.200 ± 0.154	0.0039 ± 0.0030	− 0.0172	− 1.4009^a^	− 1.5866^a^	− 1.7190^a^
	Iran	11	0	0	0	1	0	0	0	0	0	0
	Mexico	8	0	0	0	1	0	0	0	0	0	0
	Colombia	25	0.500	2	2	3	0.44 ± 0.095	0.0039 ± 0.0010	0.0017	− 0.1215^a^	− 0.6754^a^	− 0.6012^a^

*n* number of analysed sequences, *K* average number of nucleotide differences, *S* number of segregating sites, *Eta* total number of mutations, *H* number of haplotypes, *Hd* haplotype diversity, π observed average pairwise nucleotide diversity, *SD* standard deviation, *dN* rate of non-synonymous mutations, *dS* rate of synonymous mutations

^a^*P *> 0.1

^b^*P* < 0.02

^c^*P* < 0.05

^d^*P* < 0.01

^e^0.05 < *P* < 0.1

## Discussion

PvCSP is one of the leading candidates for vivax malaria vaccine. Several recent PvCSP-derived vaccines were designed as multivalent formulations or chimeric molecules in an attempt to induce protective immunity against *P. vivax* [11, 21, 22]. However, the impact of natural genetic variations in the global *pvcsp* population on vaccine efficacy remains unclear. In this study, genetic polymorphism and natural selection in Myanmar *pvcsp* and global *pvcsp* populations were comprehensively analysed. Among the three *pvcsp* allelic variants, only two allelic variants, VK210 and VK247, were identified in *P. vivax* isolates from Myanmar analysed in this study. The VK210 was the dominant allele occurring in 83.6% of Myanmar *pvcsp* population, which is consistent with previous studies reporting that VK210 is the most common allelic variant in the *pvcsp* populations from Iran, India, China, Brazil, Thailand, Bangladesh, and Azerbaijan [23–31]. Meanwhile, VK247 was more prevalent in certain regions of Colombia [32]. It has been suggested that the distribution of *Anopheles* mosquito species may affect the prevalence of VK210 and VK247 variants in endemic areas [32, 33]. In Myanmar, diverse species of *Anopheles* are distributed throughout the country, and at least 10 species including *Anopheles dirus*, *Anopheles minimus*, and *Anopheles aconitus* may transmit malaria [34]. Differences in infectivity of VK210 and VK247 variants in certain *Anopheles* species is not clear; however, the differences in adaptability of each allelic variant to mosquito vectors affects the prevalence of VK210 in Myanmar. The predominance of VK210 may also be associated with its genetic diversity. VK247 showed a lower level of genetic diversity than VK210 in the Myanmar *pvcsp* population, suggesting different polymorphisms may increase the adaptability of VK210 to different mosquito vectors.

The N-terminal non-repeat region of Myanmar *pvcsp* was relatively well-conserved. Only a few amino acid changes were identified in the latter portion of the N-terminal non-repeat region of Myanmar *pvcsp*. The RI motif (KLKQP), a cell adhesive motif exposed by proteolytic cleavage after the interaction between sporozoite and hepatocyte [8], was well conserved in both Myanmar VK210 and VK247 variants. Global VK210 variants also displayed limited polymorphism in the N-terminal non-repeat region. An alanine insertion at the end of the RI was the major variation identified in global *pvcsp*, although the prevalence of the insertion differed by geographically. Amino acid changes were also identified in global VK210 variants, but they showed uneven geographic distribution with low frequencies. The N-terminal non-repeat region of global VK247 variants was also relatively well-conserved, although uneven and minor amino acid changes were also identified. It has been shown that the invasion of malaria parasite is inhibited by antibodies directed against RI motif [35]. The highly conserved genetic characteristic of the N-terminal region of global *pvcsp* suggest that this region including RI motif may represent an attractive candidate for formulation of a PvCSP-based vaccine.

As expected, high level of genetic diversity was identified in the CRR of Myanmar *pvcsp*. Both VK210 and VK247 variants showed great genetic diversity in CRR resulting in 118 and 23 different haplotypes for VK210 and VK247, respectively. Insertions and deletions of nonapeptide sequences probably resulted from either sexual recombination during meiosis or intrahelical strand slippage during mitotic DNA replication [24], which may generate novel haplotypes in Myanmar *pvcsp*. Interestingly, 47 different types of PRMs including 27 novel types were identified in the CRR of Myanmar VK210 variants. In the case of VK247 variants, a total of 8 distinct PRMs were identified, in which 5 were novel ones. These findings also suggest point mutations as one of the major factors increasing the genetic complexity of Myanmar *pvcsp*. Similar patterns of high genetic polymorphisms were also identified in the CRR of global *pvcsp* population; however, the overall diversity due to different types and repeats of PRMs was greater in the *pvcsp* from Myanmar compared with other countries. Especially, the repeated numbers of PRMs varied widely in both Myanmar VK210 and VK247 variants compared with those from other countries.

The C-terminal regions of Myanmar and global *pvcsp* also showed genetic polymorphisms. Differences in ANKKAEDA octapeptide insertion and GGNA tetrapeptide repeat motifs and amino acid substitutions throughout the region contributed to genetic diversity in Myanmar and global VK210 variants. Despite the unclear biological functions of ANKKAEDA octapeptide and GGNA tetrapeptide in VK210 variants, they may also be generated via intrahelical recombination, and parasites carrying the ANKKAEDA octapeptide show a high degree of delayed infections [11]. Similar to VK210 variants, ANKKAGDAG octapeptide and GGNA motifs were important factors contributing to genetic diversity in Myanmar and global VK247 variants. Although the frequency of octapeptide insertion and number of GGNA motifs in global *pvcsp*, both in VK210 and VK247 variants, were differed by country, no clear geographic clustering was identified. Considering the limited genetic information of the C-terminal region in global *pvcsp* in current, a further study employing larger numbers of *pvcsp* sequences in diverse geographical origins is necessary to understand the genetic nature and evolutionary aspects of the C-terminal region in global *pvcsp* population.

Analysis of the two non-repeat regions in global *pvcsp* suggests that these regions are likely to be under natural selection, which may maintain or generate genetic diversity in the global *pvcsp* population. The dN–dS values for Myanmar and Cambodia VK210 and VK247 variants were positive, implying that balancing selection might act in these regions. However, the values were 0 or negative for both VK210 and VK247 variants from other countries except the N-terminal region of South Korea VK210 and the C-terminal region of Colombia VK247. Tajima’s D and Fu and Li’s D and F values also revealed complex patterns of natural selection that were unique to *pvcsp* from each country. While *pvcsp* populations from a few countries showed no genetic diversity involving the N- and C-terminal regions, a few *pvcsp* populations showed negative Tajima’s D values, suggesting purifying natural selection. The overall trend indicated that the global *pvcsp* population decreased the genetic diversity in the two terminal non-repeat regions. Due to size polymorphisms induced by different numbers and arrangements of PRMs in CRR, a direct analysis of natural selection in CRR was not possible in this study. However, evidence supporting natural selection in the CRR have been reported [36–38]. These findings collectively suggest that complex natural selection phenomena may act on the global *pvcsp*.

Despite the remarkable reduction in malaria transmission in Myanmar during the last decades [2, 38], the high levels of genetic diversity among malaria parasites appears to persist in the country. As demonstrated in this study, the overall genetic polymorphisms of Myanmar *pvcsp* were greater than those reported from other countries analysed. Similar patterns of high genetic diversity in several polymorphic genetic markers including apical membrane antigen-1, merozoite surface protein-1 and -2, and CSP of Myanmar *P. falciparum* population have also been reported [39–42]. Despite the lack of clear insight into the high genetic diversity underlying the population decline, asymptomatic carriers may act as fundamental reservoirs contributing to malaria transmission, which provides adequate population size to maintain or generate genetic diversity of malaria parasites in Myanmar [42]. This study also had a limitation. The Myanmar *P. vivax* isolates analysed in this study were collected in restricted areas of Myanmar and, therefore, not fully representative of the nation-wide genetic diversity and population structure of Myanmar *pvcsp*. A further comprehensive study involving a larger number of *P. vivax* isolates collected from different regions in Myanmar is necessary.

## Conclusions

PvCSP is a leading candidate for vivax vaccine; however, the impact of natural genetic variations in the global *pvcsp* population on the efficacy of PvCSP-based vaccine remains unclear. This study highlights an additional level of complexity in the application of this antigen for the development of a vivax vaccine. Global *pvcsp* population revealed polymorphic characters, especially in the CRR. The size polymorphisms and the appearance of novel PRMs as well as new CRR arrays primarily contribute to the genetic diversity of global *pvcsp*. Natural selection may also be a major force affecting the genetic diversity of global *pvcsp*. The results of this study provide not only an insight into the genetic nature of global *pvcsp* but also valuable information for the development of a universal vaccine based on PvCSP. Continuous monitoring of genetic diversity of global *pvcsp* population is necessary to elucidate the polymorphic nature and evolutionary aspect of *pvcsp* in global *P. vivax* population.

## Supplementary information

**Additional file 1: Figure S1.** Schematic structure of *pvcsp*. The gene is separated into three regions; an N-terminal non-repeat region, a central repeat region (CRR), and a C-terminal non-repeat region. The CRR consists of two major repeat peptide motifs (PRMs), termed VK210 and VK247.

**Additional file 2: Table S1.** Global *pvcsp* sequences analysed in this study.

## Data Availability

The data supporting the conclusions of this article are provided within the article and its additional files. The original datasets analysed in this current study are available from the corresponding author upon request. The nucleotide sequences reported in this study have been deposited in the GenBank database under the Accession Numbers MN821829–MN821999.

## Abbreviations

CRR: Central repeat region
CSP: Circumsporozoite surface protein
dN: Rate of non-synonymous mutations
dS: Rate of synonymous mutations
H: Number of haplotypes
Hd: Haplotype diversity
*K*: Average number of nucleotide differences
PCR: Polymerase chain reaction
PRM: Peptide repeat motif
PvCSP: *Plasmodium vivax* circumsporozoite surface protein
rRNA: 18S ribosomal RNA
S: Number of segregating sites
π: Observed average pairwise nucleotide diversity
