# Supplementary material for: Genetic polymorphism and natural selection of circumsporozoite protein in Myanmar Plasmodium vivax
Source: Malar J. 2020 Sep 4;19:303. doi: 10.1186/s12936-020-03366-7 (PMC7650223; doi:10.1186/s12936-020-03366-7)
Supplement: Supplementary file 1 — Additional file 1: Figure S1. Schematic structure of pvcsp. The gene is separated into three regions; an N-terminal non-repeat region, a central repeat region (CRR), and a C-terminal non-repeat region. The CRR consists of two major repeat peptide motifs (PRMs), termed VK210 and VK247. [file 12936_2020_3366_MOESM1_ESM.pptx]

## Slide 1
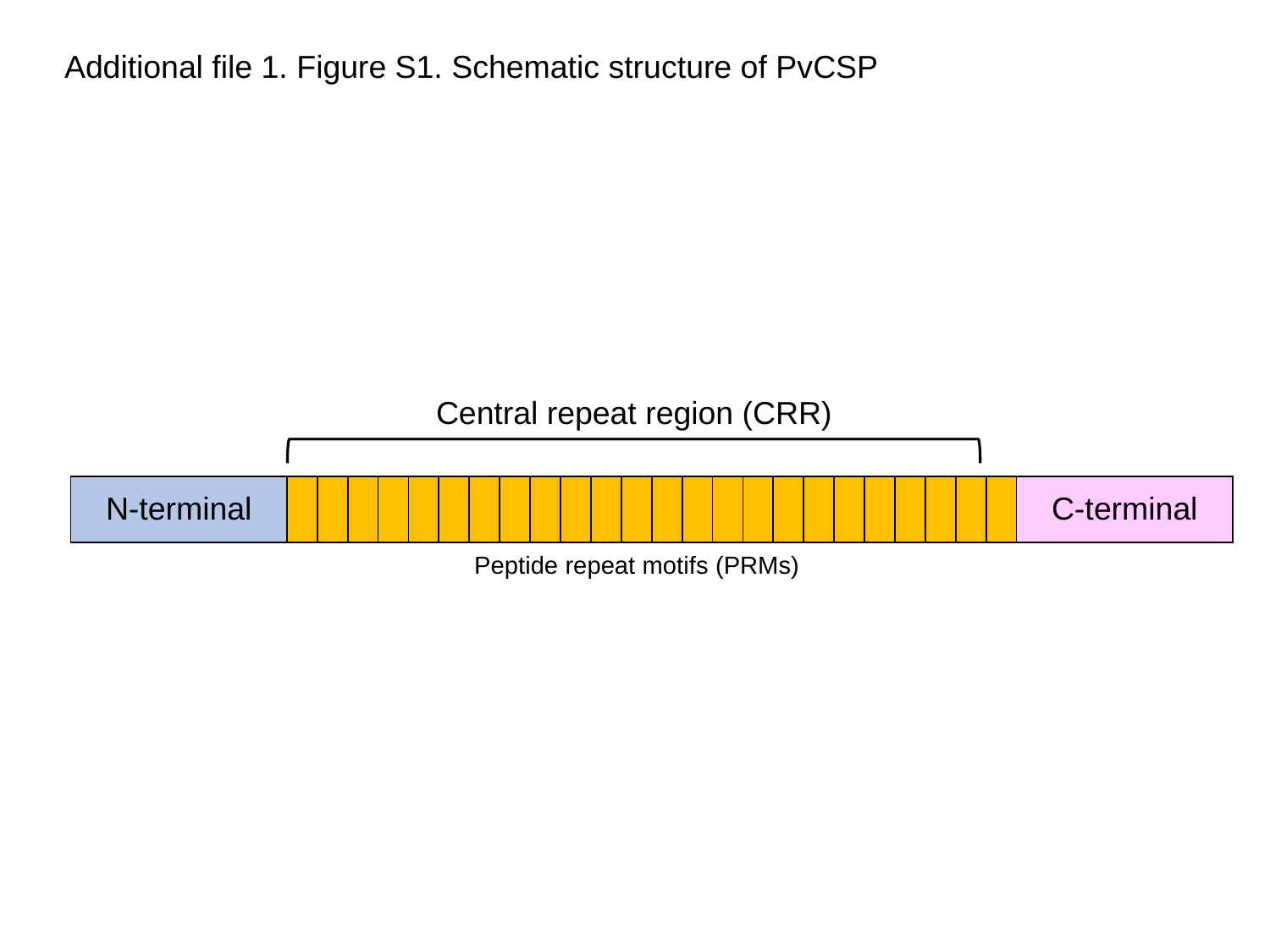

Additional file 1. Figure S1. Schematic structure of PvCSP
Central repeat region (CRR)
| N-terminal | | | | | | | | | | | | | | | | | | | | | | | | | C-terminal |
| --- | --- | --- | --- | --- | --- | --- | --- | --- | --- | --- | --- | --- | --- | --- | --- | --- | --- | --- | --- | --- | --- | --- | --- | --- | --- |
Peptide repeat motifs (PRMs)
