# Supplementary material for: Genetic polymorphism and natural selection of circumsporozoite protein in Myanmar Plasmodium vivax
Source: Malar J. 2020 Sep 4;19:303. doi: 10.1186/s12936-020-03366-7 (PMC7650223; doi:10.1186/s12936-020-03366-7)
Supplement: Supplementary file 2 — Additional file 2: Table S1. Global pvcsp sequences analysed in this study. [file 12936_2020_3366_MOESM2_ESM.docx]

**Table S1. List of global *pvcsp* sequences used in this study**

| Variant  type | Country | Number of  sequences | Genbank accession numbers |
| --- | --- | --- | --- |
| VK210 | Cambodia | 31 | JX461243, JX461245- JX461248, JX461250, JX461252, JX461254- JX461258, JX461260- JX461267, JX461269, JX461271- JX461274, JX461277- JX461279, JX461282- JX461284 |
|  | India | 79 | FJ491064-FJ491141 |
|  | Iran | 39 | KT558159- KT558161, KT558164- KT558172, KT558174, KT558175, KT558177, KT558178, KT558180, KT558182, KT558184, KT558186, KT558188- KT558195, KT558197- KT558205, KT558207, KT558208 |
|  | South Korea | 39 | DQ859734-DQ859772 |
|  | Brazil | 41 | DQ978649-DQ978689 |
|  | Mexico | 11 | JQ511263- JQ511269, JQ511271, JQ511273, JQ511276, JQ511280 |
|  | Sudan | 30 | KP162217-KP162246 |
|  | Vanuatu | 21 | AB539022, AB539023, AB539025- AB539029, AB539031- AB539038, AB539040- AB539045 |
| VK247 | Cambodia | 10 | JX461244, JX461249, JX461251, JX461259, JX461268, JX461275, JX461276, JX461280, JX461281, JX461285 |
|  | Iran | 11 | KT588162, KT588163, KT588173, KT588176, KT588179, KT588181, KT588183, KT588185, KT588187, KT588196, KT588206 |
|  | Mexico | 8 | JQ511270, JQ511272, JQ511274, JQ511275, JQ511277- JQ511279, JQ511281 |
|  | Colombia | 25 | GU339060, GU339062, GU339067, GU339070, GU339071, GU339074- GU339076, GU339061, GU339063- GU339066, GU339068, GU339069, GU339073, GU339077- GU339084, GU339086 |
